# Iron oxides nanobelt arrays rooted in nanoporous surface of carbon tube textile as stretchable and robust electrodes for flexible supercapacitors with ultrahigh areal energy density and remarkable cycling-stability

**DOI:** 10.1038/s41598-020-68032-z

**Published:** 2020-07-03

**Authors:** Yuying Ding, Shaochun Tang, Rubing Han, Sheng Zhang, Guanjun Pan, Xiangkang Meng

**Affiliations:** National Laboratory of Solid State Microstructures, Collaborative Innovation Center of Advanced Microstructures, Jiangsu Key Laboratory of Artificial Functional Materials, College of Engineering and Applied Sciences, Department of Materials Science and Engineering, Haian Institute of High-Tech Research, Nanjing University, Jiangsu, 210093 People’s Republic of China

**Keywords:** Materials for devices, Energy storage

## Abstract

We report a significant advance toward the rational design and fabrication of stretchable and robust flexible electrodes with favorable hierarchical architectures constructed by homogeneously distributed *α*-Fe_2_O_3_ nanobelt arrays rooted in the surface layer of nanoporous carbon tube textile (NPCTT). New insight into alkali activation assisted surface etching of carbon and in-situ catalytic anisotropic growth is proposed, and is experimentally demonstrated by the synthesis of the Fe_2_O_3_ nanobelt arrays/NPCTT. The Fe_2_O_3_/NPCTT electrode shows excellent flexibility and great stretchability, especially has a high specific areal capacitance of 1846 mF cm^−2^ at 1 mA cm^−2^ and cycling stability with only 4.8% capacitance loss over 10,000 cycles at a high current density of 20 mA cm^−2^. A symmetric solid-state supercapacitor with the Fe_2_O_3_/NPCTT achieves an operating voltage of 1.75 V and a ultrahigh areal energy density of 176 µWh cm^−2^ (at power density of 748 µW cm^−2^), remarkable cycling stability, and outstanding reliability with no capacity degradation under repeated large-angle twisting. Such unique architecture improves both mechanical robustness and electrical conductivity, and allows a strong synergistic attribution of Fe_2_O_3_ and NPCTT. The synthetic method can be extended to other composites such as MnO nanosheet arrays/NPCTT and Co_3_O_4_ nanowire arrays/NPCTT. This work opens up a new pathway to the design of high-performance devices for wearable electronics.

## Introduction

The urgent demand for solving the future energy crisis and increasing environmental concerns has motivated the development of high-performance green energy conversion and storage devices. Among various emerging energy storage technologies, supercapacitors (SCs), which bridge the gap between conventional dielectric capacitors (with high power output) and batteries (with high energy storage), are currently attracting significant attention due to their high power density, fast charge–discharge rate, and long cycle life^1–3^. However, one of the key challenges that limits SCs’ practical applications is to increase their areal/volumetric energy density to the value approaching to or even exceeding that of conventional batteries without sacrificing other performance^4–7^. On the other side, next-generation electronics are expected to be flexible and wearable^8–10^, it is thus highly required to develop flexible advanced electrode materials with favorable architectures allowing large porosity, high conductivity, and strong mechanical stability. In addition, using environmentally friendly a solid-state electrolyte with fast ion transport is of great importance.

Based on electrode materials and charge storage mechanism, SCs are classified as electrical double layer capacitors (EDLCs) and pseudocapacitors. The main electrode materials for EDLCs are carbonaceous materials, and major challenge is low energy density. Pseudocapacitors hold great promise for improving energy density based on the use of pseudocapacitive redox active materials by fast and reversible surface redox reactions at or near the electrode/electrolyte interfaces^11,12^. Transition metal oxides, such as Fe_*x*_O_*y*_, RuO_2_, Co_3_O_4_, and MnO_*x*_ have been investigated as electrode materials for pseudocapacitive SCs. Iron oxides (Fe_*x*_O_*y*_) materials possess a high hydrogen evolution potential in aqueous solution, making them promising candidates for the negative electrode in SCs^13–18^. Among the iron compounds, hematite *α*-Fe_2_O_3_ has attracted increasing intention since it is of technological and scientific importance for applications like anode in Li-ion batteries, dye solar cells, alcohol-sensing, H_2_S decomposition and water splitting^13,18,19^. More importantly, *α*-Fe_2_O_3_ is one of the most promising SC materials because of its ideal theoretical specific capacitance (3,625 F g^−1^)^20^, wide operating potential, low cost, and abundant availability. However, its poor electronic conductivity (being–10^–14^ S cm^−1^)^21,22^ severely hinders the charge storage capability, which results in that their actual specific capacitance (120–300 F g^−1^) is far from the theoretical value^13–20^, considerably low capacitance retention at high current densities, and limited electrochemical stability. An effective strategy to improve the electrical conductivity is to develop hybrid materials by combining metal oxides/hydroxides with conductive carbonaceous materials such as carbon nanotubes^23^, carbon black^24^, and graphene^25,26^. Most of the reported composites are powder-like and the use of binders leads to poor utilization of active materials. This issue can be addressed by developing binder-free, hierarchically porous electrodes with an enhancement in power density and rate capability because the porosity enables fast electrolyte interaction and ion transport. Much effort has been focused on growing arrays of nanomaterials with well-defined shapes like nano-needles^27^, nanowires^28^, nanotubes^29^, nanosheets^6,27^, and tetsubo-like^19^ on a on current collector. However, a weak interfacial interaction between the grown nanostructures and carbon textiles’ ligaments with very smooth and high-curvature surfaces (carbon tubes or carbon fibers) results in that the electrochemical stability is unsatisfying^30,31^ because the grown materials usually separate from a ligament during charge–discharge^32^.

As for flexible substrates, a 3D conductive carbon scaffold is pursuing because it can provide interconnected network in 3D space and storage charges due to electric double-layer capacitive (EDLC) mechanism^33^, but the capacitance of most experimentally synthesized carbon materials is typically below 300 F g^−1^^10^. Carbon textiles from carbonization of cotton textiles were demonstrated to have excellent mechanical flexibility, strength, and electrical conductivity^34^, and thus are expected to be excellent flexible substrates for growing other active materials. Nevertheless, the reported carbon textiles based composites are usually synthesized by two-step procedures including the first carbonization and subsequent surface deposition of nanostructures^28,35^. To the best of our knowledge, a study of one-step, highly-efficient, and scalable synthesis of transition metal oxides like Fe_2_O_3_ nanobelt arrays rooted in surface layer of carbon textiles for SCs has not been reported to date.

Herein, we report the rational design and fabrication of a stretchable and robust flexible electrode with favorable hierarchical architectures, in which homogeneously distributed *α*-Fe_2_O_3_ nanobelt arrays vertically grown and rooted in the surface layer of a nanoporous carbon tube textile (NPCTT). New insight into alkali activation assisted surface etching and in-situ catalytic growth is proposed. This general method can be extended to other composites such as MnO nanosheet arrays/NPCTT and Co_3_O_4_ nanowire arrays/NPCTT. The Fe_2_O_3_/NPCTT has excellent flexibility and stretchability, shows a specific areal capacitance reaching 1846 mF cm^−2^ at 1 mA cm^−2^ and remarkable cyclic stability (only 4.8% capacitance loss over 10,000 cycles at 20 mA cm^−2^). These are ascribed to such unique architecture that can offer numerous channels for rapid ion diffusion and high electrical conductivity. Also, Fe_2_O_3_ nanobelts strongly coupled on the interconnected and the conductive carbon tube network, which much improves mechanical stability of the whole hybrid structure. The application of the Fe_2_O_3_/NPCTT electrode in flexible solid-state SCs has been demonstrated to achieve ultrahigh areal energy densities and to retain normal electrochemical performance under various bending or twisting states.

## Results

### Synthesis and characterization of Fe_2_O_3_/NPCTT composite electrode

**Figure 1:**
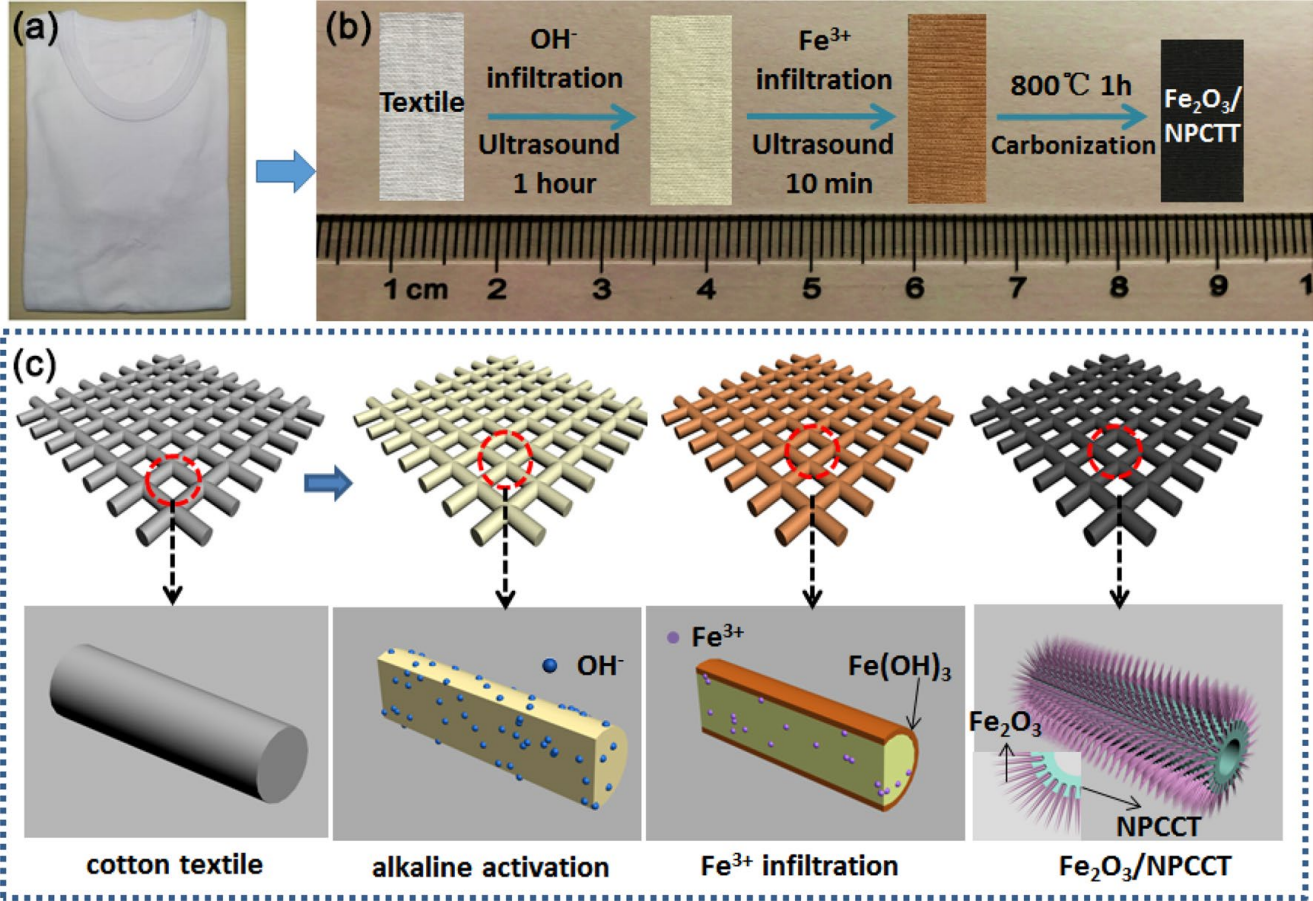
(**a**) A photograph of a commercially available cotton T-shirt, (**b**) digital photos showing color change of a piece of pristine cotton textile cut from the T-shirt before (left) and after alkaline activation, Fe^3+^ infiltration, and the resulting Fe_2_O_3_ nanobelts/NPCTT, (**c**) schematic illustrating formation process of the Fe_2_O_3_/NPCTT. The lower schematic diagrams show detailed surface layer changes of a single fiber in the textile as well as the grown nanostructures’ features at different stages.

The preparation procedures of the Fe_2_O_3_-nanobelt arrays/NPCTT are described in detail in the Experimental Section (Supporting Information). The Fe_2_O_3_/NPCTT composite was prepared using a low-cost commercially available white cotton T-shirt (as shown in a photograph of Fig. 1a) as the starting material. Figure 1b presents digital photos showing a color change during main three-step fabrication process, which starts from a piece of pristine cotton textile before (left) and after alkaline activation, to Fe^3+^ infiltration (the second step), and high-temperature carbonization with simultaneous in-situ catalytic growth. The pristine cotton textile has a size of 3 cm length × 1 cm width. Subsequent alkaline activation (OH^−^ infiltrating), Fe^3+^ ion infiltrating, and high temperature carbonization turned the white textile into yellowish (the second one from left), reddish brown (the third one), and finally deep black, respectively. Figure 1c schematically illustrates formation process of Fe_2_O_3_ nanobelt arrays rooted in the surface layer of each carbon tube (CT) of NPCTT. The lower schematic diagrams show the corresponding surface change of a single fiber in the textile as well as the grown nanostructures’ features at different stages. It is emphasized that the alkaline activation (OH^−^ infiltration) of cotton textile is the key step to obtain a desirable nanostructure. Owing to strong water absorption and conservation ability of cotton, it is expected that OH^−^ ions are embedded in the surface layer of each cotton fiber. After that, the entry of hydroxyl provides many active sites in the surface layer of the solid cotton fibers. If alkaline activation is not used with other reaction parameters remained, the formed CTs in the carbonized textile will have a smooth surface, especially nothing is observable on the surface of each CT.

**Figure 2:**
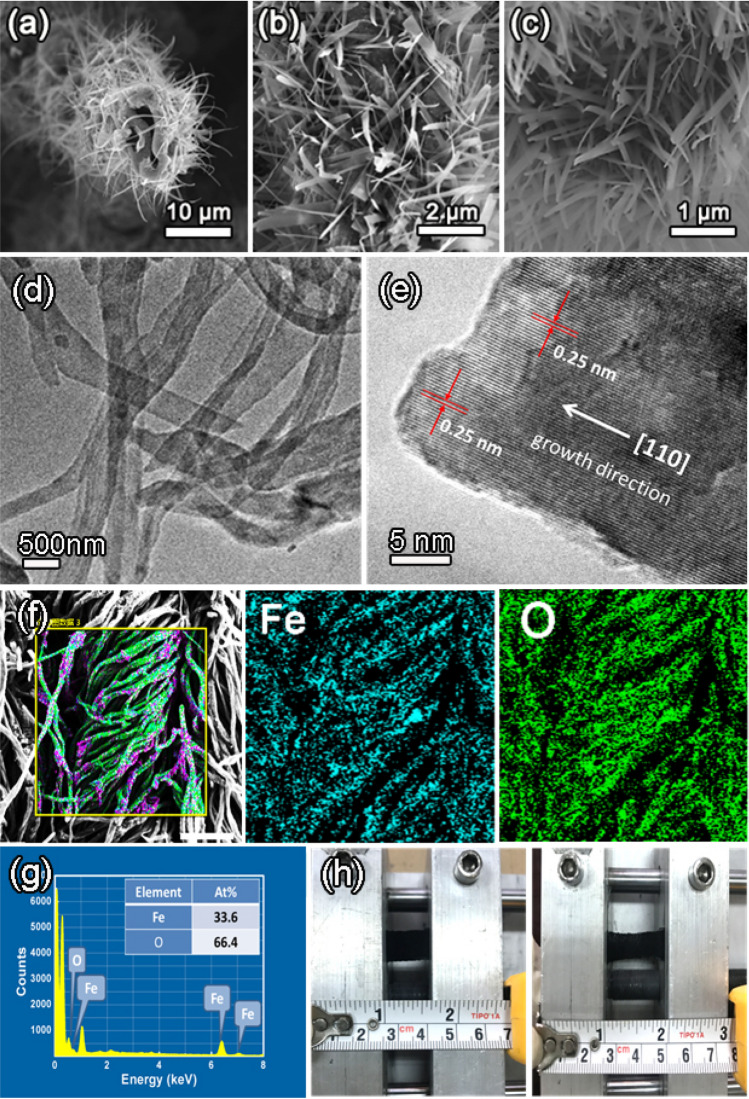
(**a**) low- and (**b**, **c**) high-magnification SEM images of a typical Fe_2_O_3_/NPCTT; (**d**) Low-magnification TEM image of several nanobelts separated from the CTs, and (**e**) HRTEM image showing a single nanobelt. (**f**) SEM-mappings of Fe and O elements recorded from a large area; (**g**) EDX spectrum shows these elements’ characteristic peaks, proving co-existence of Fe and O elements with their atomic percentages; (**h**) photographs of a Fe_2_O_3_/NPCTT electrode under stretching state, indicating its excellent flexibility and stretchability.

From a cross-section SEM image recorded from a single broken Fe_2_O_3_-nanobelt arrays/NPCTT, as shown in Fig. 2a, one can see that the resulting typical product has a core–shell structure with hollow carbon tube as core and many grown nanobelts as shell. The wall thickness of a single tube (core) is about 1 µm. The newly formed nanobelts cover the tube outer surface, separate from each other and form homogeneously distributed arrays radially along the entire length of a tube. A front view SEM image at a higher magnification (Fig. 2b) reveal the belt-like morphology of the grown nanostructures and their spatial distribution around the tube.

Further magnification SEM (Fig. 2c) reveals that the nanobelts have smooth surfaces with a width ranging from 200 to 400 nm and lengths larger than 5 μm. It should be emphasized that the nanobelts’ array has a strong binding force with the nanoporous CTs due to the unique structure, even after ultrasound irradiation for 1 h at a power of 100 W, they are still standing on the CTs. The transmission electron microscopy (TEM) image in Fig. 2d clearly verifies the belt-like shape, revealing smooth surface with width of around 300–400 nm for the nanobelts. Their thickness looks to be uniform. The morphology and sizes are consistent with the SEM results. As shown in Fig. 2e, one can see from a high-resolution TEM (HRTEM) image that a nanobelt is single-crystalline. The measured interplanar spacing of 0.25 nm for the well-defined lattice fringes matches well with the *α*-Fe_2_O_3_ (110) planes (JCPDs no. 39-1346). Since the thin nanobelt is lying on the carbon film supported by a copper mesh, the growth direction happens to be parallel to the lattice fringes (no rotation between them). That is to say, based on the HRTEM analyses, it is revealed that the growth direction along the long axis of *α*-Fe_2_O_3_ belts is determined to be [110].

This unique nanoarchitecture was also demonstrated by the energy-dispersive X-ray spectroscopy (EDS) elemental mappings. The elemental mappings (Fig. 2f) recorded from a large designated area (as circled in a low-magnification SEM) prove co-existence of Fe and O elements, and reveals their homogeneous distribution along the long carbon tubes in a textile. Based on the EDS analysis (Fig. 2g), the strong characteristic peaks correspond to two main elements Fe and O, and their atomic percentages are 33.4% and 66.6%, respectively. The Fe/O atomic ratio is thus 1:2, which is slightly larger than the standard atomic ratio 2:3, which is probably resulted from nanosized Fe(0) embedded in the surface layer of the carbon tubes, which will be discussed later. Moreover, it is remarkable that the composite textile electrode can be stretched to 1.5 times of its original length. Figure 2h shows the Fe_2_O_3_/NPCTT electrode under being stretched state, indicating its high flexibility, which promises its potential applications for stretchable all-solid-state energy storage devices.

**Figure 3:**
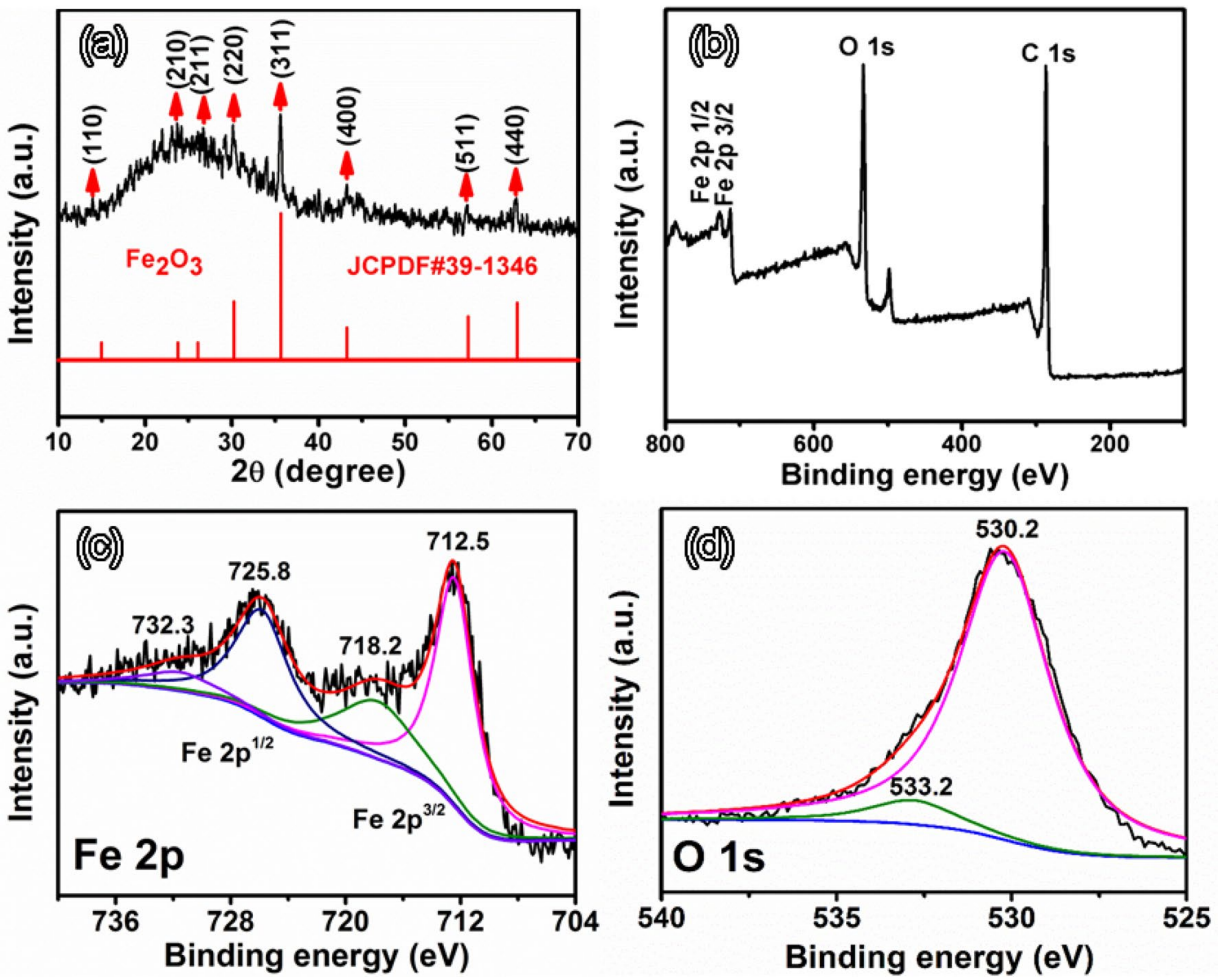
(**a**) XRD pattern, (**b**) XPS survey, high-resolution XPS spectra of (**c**) Fe 2*p*, and (**d**) O 1*s* recorded from the typical Fe_2_O_3_/NPCTT.

As shown in the XRD pattern (Fig. 3a), except for a strong and wide peak for carbon, other diffraction peaks correspond to spinel α-Fe_2_O_3_. The diffraction peaks at 14.9°, 23.8°, 26.1°, 30.2°, 35.6°, 43.2°, 57.2° and 62.9° can be indexed to the (110), (210), (211), (220), (311), (400), (511) and (400) of hematite *α*-Fe_2_O_3_, respectively (JCPDs no. 39-1346). No peaks corresponding to the Fe (0) are found in the pattern. Figure 3b–d show X-ray photoelectron spectroscopy (XPS) spectra recorded from the sample. The strong signals of C, O, and Fe elements are observed in the XPS survey (Fig. 3b). In the high resolution Fe2*p*3/2 spectrum (Fig. 3c), two distinct peaks at the binding energies of 712.5 eV for Fe 2*p*3/2 and 725.8 eV for Fe 2*p*1/2 with two shake-up satellites at 718.2 and 732.3 eV can be observed. These binding energies and the distance between the 2*p*3/2 and 2*p*1/2 peaks are both well consistent with those reported for Fe_2_O_3_ in the literature^16,17^. The high-resolution O1*s* spectrum could be deconvoluted into two different components at the binding energies of 530.2 and 533.2 eV, representing the existence of the lattice oxygen and physically adsorbed oxygen (H_2_O), respectively (Fig. 3d)^36^. The lattice oxygen could be attributed to the oxygen bonded to Fe in a trivalent state, and the adsorbed water was detected by thermogravimetric analysis (TGA) during temperature rise in low temperature range. Therefore, combined with above-mentioned SEM–EDS elemental mappings and Fe/O atomic ratio, the XPS and XRD analysis results further demonstrate the generation of *α*-Fe_2_O_3_/C composite.

TGA was performed to accurately determine the weight percentage of Fe_2_O_3_ in the composite. TGA curve (Figure S1, Supporting Information) shows a first weight reduction from room temperature to 100 °C, which is attributed to removal of surface adsorbed water. Then a slight weight decrease (16% weight loss) is observed from 100 to 330 °C. As the temperature further rises, it shows that the most significant changes take place from 340 to 500 °C, during which a little Fe catalyst facilitated oxidation of carbon takes place. After that point, a continuous weight loss from 350 to 500 °C originates from the combustion of carbon. When the temperature is higher than 500 °C, no weight loss happens any longer. Based on the weight loss, the loading percentage of Fe_2_O_3_ in the typical composite material is 7.79 wt%.

## Discussion

### Effects of alkali activation and the Fe^3+^ concentration

As mentioned above, alkali activation process of cotton cloth is the key stage. The generation of nanoporous in surface layer of CTs can be confirmed by a control experiment. As shown in a low-magnification SEM image (Figure S2b, Supporting Information), an original cotton textile consists of tightly compacted cotton fiber bundles, a single fiber has a diameter of ~ 12 μm and a smooth surface. In the carbonization process, alkali activation makes each carbon tube porous due to the generation of gas during the thermal pyrolysis of cotton cellulous. Without alkali immersion, cotton fibers become smooth carbon tubes with their diameters reduced to ~ 8 μm due to being hollowed out during carbonization (Figure S2c). After alkali immersion, individual carbon tube has rough and nanoporous surface, as confirmed by high-magnification SEM images where many small holes are observable, as shown in Figure S2e and S2f. Obviously, the surface of the resulting carbon tube was etched due to alkaline activation (compared with S2d). This is consistent with previously repored alkali activation leading to porous carbon^37,38^. At the same time, the carbon tubes separate from each other leading to loose bundles with larger inner pace. Without the alkali activation process of cotton cloth, the grown nanostructures (e.g., nanoparticle coating, Figure S3a, Supporting Information) easily separate from the smooth surfce of carbon tubes due to a very weak binding beteen them. Even a uniform coating of nanowire arrays will be partly removed from the surface after ultrasond irradiation for only 10 min at a power of 100 W (Figure S3b). Commercial carbon cloth is similar to the carbon tubes without the alkali activation, the smooth surface makes grown nanostructures to be easily separated due to weak binding force.

The morphology of grown Fe_2_O_3_ is highly dependent on Fe^3+^ concentration (defined as *C*_Fe_^3+^). At a low *C*_Fe_^3+^ of 5 mM, many short nanobelts form on each carbon tube (Figure S4a, Supporting Information). When *C*_Fe_^3+^ is doubled (Figure S4b), beside many nanobelts, a few large particles are observable. Comparing with the optimal *C*_Fe_^3+^  = 20 mM product (Fig. 2a–c), the number density of nanobelts is relatively smaller. At a higher *C*_Fe_^3+^  = 30 mM, nanobelts with a sparser distribution are obtained (Figure S4c). Their growth in the surface layer is confirmed. Increasing *C*_Fe_^3+^ to 50 mM (Figure S4d), nanobelts look much wider and a relatively smooth coating formed. This is because a low *C*_Fe_^3+^ allows a sufficient time for anisotropic crystal growth leading to well-defined nanobelts, while a high concentration results in a continuous layer-by-layer deposition on the carbon tubes’ surfaces.

### Formation mechanisms of Fe_2_O_3_ nanobelt arrays

**Figure 4:**
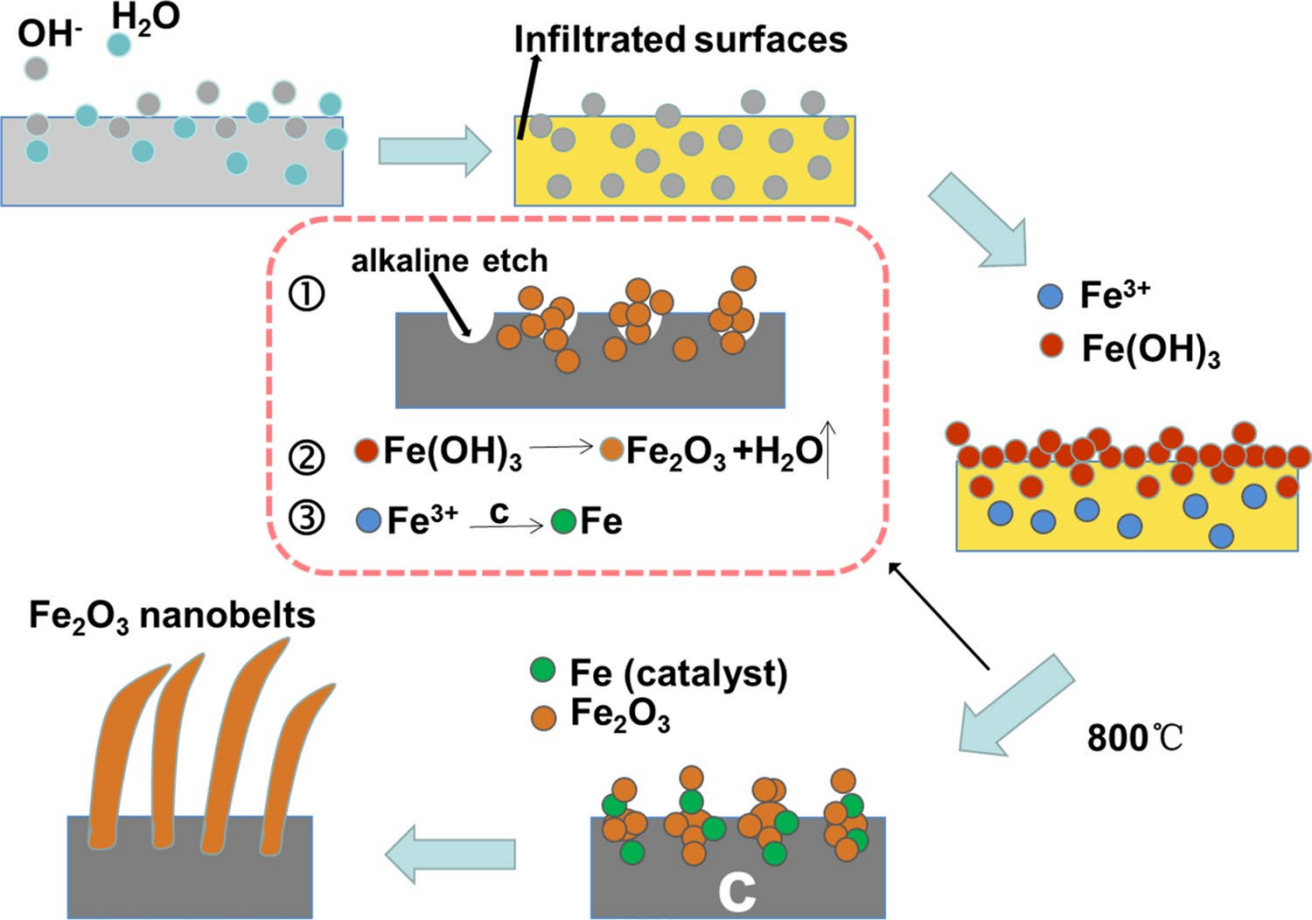
Schematic illustrating in-situ catalytic growth of Fe_2_O_3_ nanobelts during high-temperature carbonization of an alkali activated cotton leading to Fe_2_O_3_/NPCTT.

Based on the discussion above, formation mechanism of nanobelt arrays rooted in the surface layer of activated CTs is discussed as follows. Figure 4 schematically illustrates in-situ catalytic growth of Fe_2_O_3_ nanobelts during carbonization of an alkali activated cotton. After an immersion in a NaOH solution, a whole cotton fiber in the textile was infiltrated by OH^−^ ions because cotton is known to have a strong water absorption ability. Then these OH^−^ ions locate in the interior of the cotton fiber during vacuum drying. The entry of OH^−^ provides many sites where newly entered Fe^3+^ ions react with OH^−^ via Fe^3+^ + 3OH^−^ = Fe(OH)_3_. However, the formed Fe(OH)_3_ will block the channels, which result in that Fe(OH)_3_ only forms in the surface layer of a fiber while those Fe^3+^ ions embedded in the deeper positions are remained. Subsequently, three main possible processes happen simultaneously, as shown in the central dashed frame. (1) the cotton fibers are etched by the remained OH^−^ ions inside during carbonization, which leads to nanoporous surfaces. The nanopores not only provides larger specific surface area to facilitate electrolyte ion diffusion but also contributes to sufficient sites leading to in-situ growth. (2) Fe(OH)_3_ is decomposed into Fe_2_O_3_ based on an equation 2Fe(OH)_3_ = Fe_2_O_3_ + 3H_2_O. In this process, not only the composition change from Fe(OH)_3_ to Fe_2_O_3_ but also an *in-situ* growth of Fe_2_O_3_ occurs. (3) Under the Ar-gas protected conditions, the decomposition temperature of Fe(OH)_3_ gets much higher, and those remained Fe^3+^ ions could be transformed into many embedded Fe clusters due to the reduction of carbon at a high temperature^39^. Therefore, the Fe clusters serve as a catalyst for inducing anisotropic *in-situ* growth of Fe_2_O_3_, which leads to that Fe_2_O_3_ nuclei in the surface layer grow into single-crystalline nanobelts along the [110] direction, which are standing and rooted in the surface layer of a nanoporous carbon tube. This is similar to the Fe catalyzed anisotropic growth of low-dimensional inorganic nanomaterials^40,41^. Note that the amount of Fe (0) is too low to be dectectable by XRD, and also cannot be detectable by XPS that is a surface analysis technique that collects information on several nanometers depth away from the outer surface of samples. The already formed nanochannels in the surface layer during carbonization provides space for the anisotropic growth staring from the nuclei. As a result, Fe_2_O_3_ nanobelt arrays rooted in the surface layer of the activated nanoporous carbon tubes formed.

**Figure 5:**
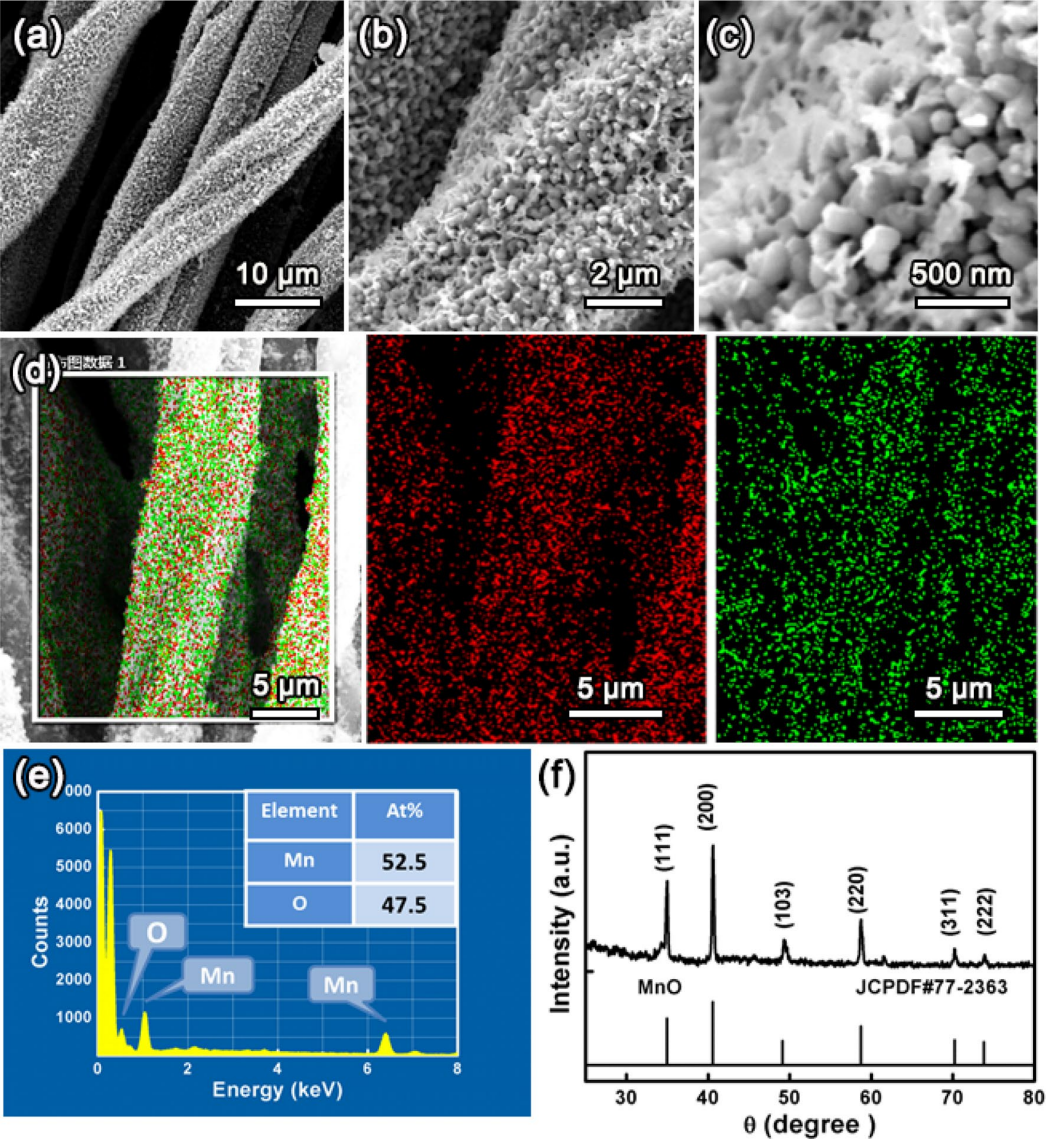
(**a**–**c**) SEM images at different magnifications of typical MnO/NPCTT product. (**d**) SEM-mapping of elemental Mn and O, (**e**) EDS spectrum with the inset showing atomic percentages of Mn and O, and (**f**) XRD pattern.

More importantly, our proposed one-step method is powerful and applicable to synthesis of other transition metal oxides/NPCTT flexible electrodes. This indicates that the present concept is facile and potentially represents a general strategy that can be extended to synthesize other transition metal oxides nanostructure arrays/NPCTT. For instance, MnO nanosheets rooted in the surface layer of each nanoporous CT in NPCTT was obtained. Each CT has a rough surface because of its being covered by homogeneously distributed nanostructures (Fig. 5a). SEM images at higher magnifications (Fig. 5b, c) indicate that the newly grown nanosheets are standing on the surface. No any aggregate is observed although they are close to each other, forming a porous and rough covering with strong binding with the CTs. Nanobelts and nanosheets are both 2D nanostructures from anisotropic growth. For different materials, the crystal growth kinetics and the resulting morphogly are much different. As a comparison, the anisotropic growth assisted by Fe (0) catalyst has a stronger tendency, resulting in generation of nanobelts with a large longth/width ratio, while the solw growth leads to thick MnO nanosheets with a denser distribution.

Elemental SEM-mappings recorded from a single composite tube (Fig. 5d) reveal that Mn (red) and O (green) elements distribute continuously throughout the whole tube. The characteristic peaks in the EDS spectrum (Fig. 5e) further prove the coexistence of Mn and O elements with their atomic percentages being 52.5 and 47.5 at.%, respectively. The Mn/O molar ratio is thus about 1:1. In the XRD pattern (Fig. 5f, Supporting information), obvious diffraction peaks are all indexed to crystal planes of MnO (JCPDS 77-2363). In high-resolution spectrum of Mn2*p* (Figure S5, Supporting Information), two obvious distinct peaks correspond to the 2*p*3/2 and 2p1/2 of Mn. An energy separation of 11.7 eV between the Mn 2*p*3/2 and Mn 2*p*1/2 peaks demonstrates that Mn element exists in the form of Mn^2+^, which is in agreement with previous report^42^. Based on the XPS analysis, the atomic molar ratio of Mn and O is about 1:1, which is consistent with the EDS result. It is known that element Mn has several different valence states in its oxides and usually exists as MnO_2_. The generation of MnO (not MnO_2_) in our product is because the strong reduction ability of carbon at high temperatures leading to the Mn element to be at a low valence state.

Similarly, in-situ growth of arrays during high-temperature carbonization is also observed in the synthesis of Co_3_O_4_ nanowire arrays/NPCTT. Figure S6 (Supporting Information) shows SEM images of the typical Co_3_O_4_/NPCTT product. Each carbon tube’s surface looks rough covered with grown nanostructures. A locally magnified SEM (see the inset) shows that arrays of nanowires with a diameter of 80–100 nm are obtained, SEM-mappings of elemental O and Co distribution along one composite tube (the right images), confirming the synthesis of Co_3_O_4_. Since the concentration dependent morphology and size of grown nanostructure arrays are adjustable, this indicates that the method offers the possibility of engineering transition metal oxides with tunable nanostructure and composition.

### Electrochemial performance

**Figure 6:**
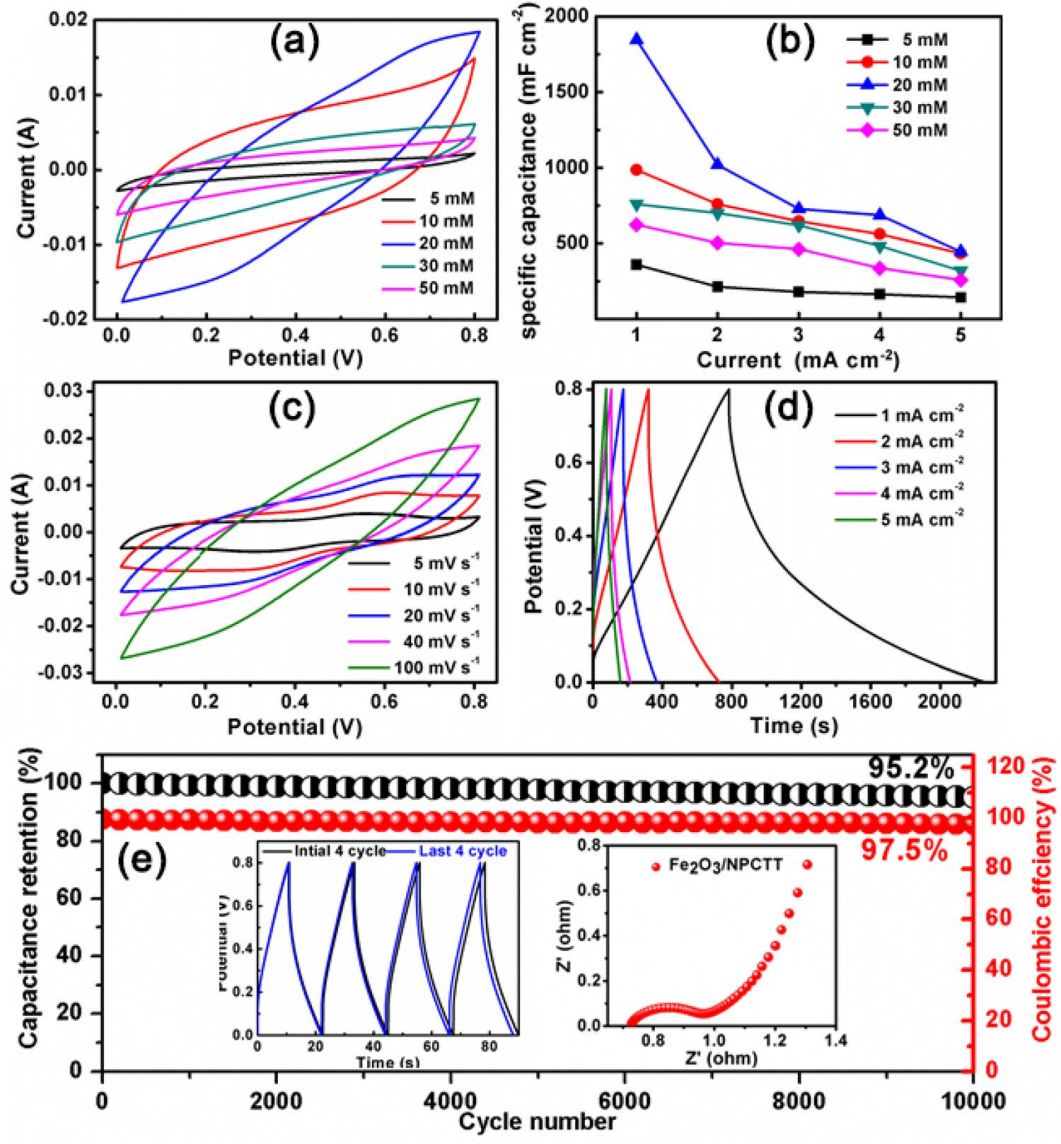
(**a**) CV curves of of the electrodes obtained with different *C*_Fe_^3+^ at a scan rate of 10 mV s^−1^, and (**b**) the corresponding Ca values of these electrodes at different current densities. (**c**) CV measured at different scan rates and (**d**) GCD curves at different current densities of the typical Fe_2_O_3_/NPCTT electrode. (**e**) Cycling performance and Coulombic efficiency at a high current density of 20 mA cm^−2^ with insets showing the initial and last four cycles and EIS curve of Fe_2_O_3_/NPCCT.

Figure 6a shows the CV curves of the electrodes obtained with different *C*_Fe_^3+^ at a scan rate of 10 mV s^−1^. The area integrated under the curves changes with the *C*_Fe_^3+^, which indicates that the largest specific capacitance can be obtained because the integrated area corresponds to the overall charge released during the discharge process. The corresponding galvanostatic charge–discharge (GCD) curves (all at 1 mA cm^−2^) are shown in Figure S7 of Supporting Information where the capacitance (discharge time) maximization is consistent with that from the CV result. According to the discharge curves, the corresponding *C*_a_ values of these electrodes at different current densities are shown in Fig. 6b. Obviously, at a given current density, increasing *C*_Fe_^3+^ leads to that the specific capacitance increases initially and then decreases rapidly. The specific capacitances are all maximal at the *C*_Fe_^3+^ = 20 mM at different current densities, which further confirms that the nanostructure optimization (Figure S4) and strong synergy between are achieved simultaneously at that point.

Figure 6c shows CV curves of the optimal Fe_2_O_3_/NPCTT electrode at different scan rates. The CV curves’ area increases with the scan rate. A pair of obvious redox peaks are observed in the CV curves. A redox couple of Fe^3+^/Fe^2+^ are suggested for Fe_2_O_3_ in the potential window of 0–0.8 V, thus the voltage window of 0–0.8 V was selected for the electrochemical measurements of the electrodes in three-electrode cell configuration. Figure 6d shows GCD curves of the electrode at various current densities. They are symmetric with linear triangular shapes, indicating ideal capacitive behavior. Based on the discharge curves, specific areal capacitances (*C*_*a*_) the Fe_2_O_3_/NPCTT electrode are 1846, 1,020, 728, 545, and 446 mF cm^−2^ at 1, 2, 3, 4, and 5 mA cm^−2^, respectively. These areal capacitances are several orders of magnitude of commercially available carbon fiber cloth with a very low areal capacitance^43^ (only 13 mF cm^−2^ at 1 mA cm^−2^)^44^. The areal capacitance *C*_a_ value of 1846 mF cm^−2^ (at 1 mA cm^−2^) is more than twice of that for molten-NaNH_2_ activated carbon cloth (744.5 mF cm^−2^ at 1 mA cm^−2^)^45^, ten times of that for N-doped carbon nanotubes (180 mF cm^−2^ at 0.5 mA cm^−2^)^46^ and 14 times of that for N-doped carbon cloth (136 mF cm^−2^ at 0.5 mA cm^−2^)^47^. This implies that the enhanced capacitance is mainly attributed to the significant contribution of pseudocapacitive Fe_2_O_3_ nanobelt arrays. This *C*_a_ value is two orders of magnitude higher than those of previously reported values for Fe_2_O_3_ based composite electrode materials, such as Graphene-wrapped Fe_2_O_3_ nanowire networks (3.3 mF cm^−2^)^48^. Also, this value is higher than three times of those for Fe_2_O_3_ nanorods@graphene foam on Ni foam (572 mF cm^−2^)^49^, Fe_2_O_3_ nanorods@NiO nanosheets on carbon cloth (557 mF cm^−2^)^15^, and Fe_2_O_3_ nanoparticles@graphene foam-CNT (470.5 mF cm^−2^)^50^. The data for the performance comparison are shown in Table S1^15,27, 44–50^.

The superior capacitive performance of Fe_2_O_3_/NPCCT can be attributed to the following three aspects: (1) The unique structure of Fe_2_O_3_ nanobelts rooted in surface layer of CTs greatly reduces the contacting resistance between them, which could effectively improve electrical conductivity facilitating electron transport and accelerate ion diffusion, resulting in high storage capability. (2) A strong synergistic attribution of pseudocapacitive Fe_2_O_3_ by the redox couple of Fe^2+^/Fe^3+^ and EDLC NPCTT by surface adsorption of electrolyte ions, both improve the overall performance. The small sizes in thickness of Fe_2_O_3_ nanobelts ensure many accessible active sites leading to enhanced redox reactions. (3) The highly porous nanobelts array architecture can provide abundant electro-active sites for both EDLC and redox reactions to substantially enhance the specific capacitance of Fe_2_O_3_ due to improved adsorption of ions and the effectively enhanced utilization ratio of electrode materials; Many advantages such as richness in accessible electro-active sites, short ion transport pathways, and high electron collection efficiency can be achieved.

Long-term cycling stability of the Fe_2_O_3_/AACT electrode was also evaluated (Fig. 6e). Remarkably, 95.2% of initial capacitance of the electrode is remained after 10,000 charge–discharge cycles at a high current density of 20 mA cm^−2^. The inset shows the initial and the last several periods (the left inset), revealing a regular charging–discharging behavior. The Coulombic efficiency *η* is as high as 97.5% after 10,000 cycles. This indicates remarkable cycling stability of the electrode, much better than other composite electrodes especially using commercial carbon cloth as substrates, such as Fe_2_O_3_ nanorods@NiO nanosheets on carbon cloth (96.2% after 3,000 cycles)^15^, Graphene-wrapped Fe_2_O_3_ nanowire networks (78.2% after 5,000 cycles)^48^, and porous Fe_2_O_3_ nanosheets on carbon fabric (93% capacitance after 4,000 cycles) (Table S1)^44^. After the cycle measurements, the Fe_2_O_3_ nanobelt arrays were still well attached on the CTs (Figure S8, Supporting Information), confirming the strong bonding between Fe_2_O_3_ nanobelt and each CT. The Nyquist plots of the electrical impedance spectroscopy (EIS) measurements are presented in the right inset of Fig. 6e. A semicircle at high frequencies corresponds to the Faradic charge-transfer resistance (*R*_ct_). It can be seen that the Fe_2_O_3_/NPCCT electrode has a smaller charge-transfer resistance (*R*_ct_ = 0.52 Ω) and solution resistance (*R*_s_ = 0.93 Ω), these values are smaller than those for pure metal oxides, such as NiO^51^ and Co_3_O_4_^52^, which results from the composite being a better electrically conducting network. Therefore, the outstanding cycling life should be attributed to the unique structure of Fe_2_O_3_ nanobelts rooted in the NPCTT as the dominating factor endowed the electrode with strong mechanical stability and excellent electrical conductivity.

**Figure 7:**
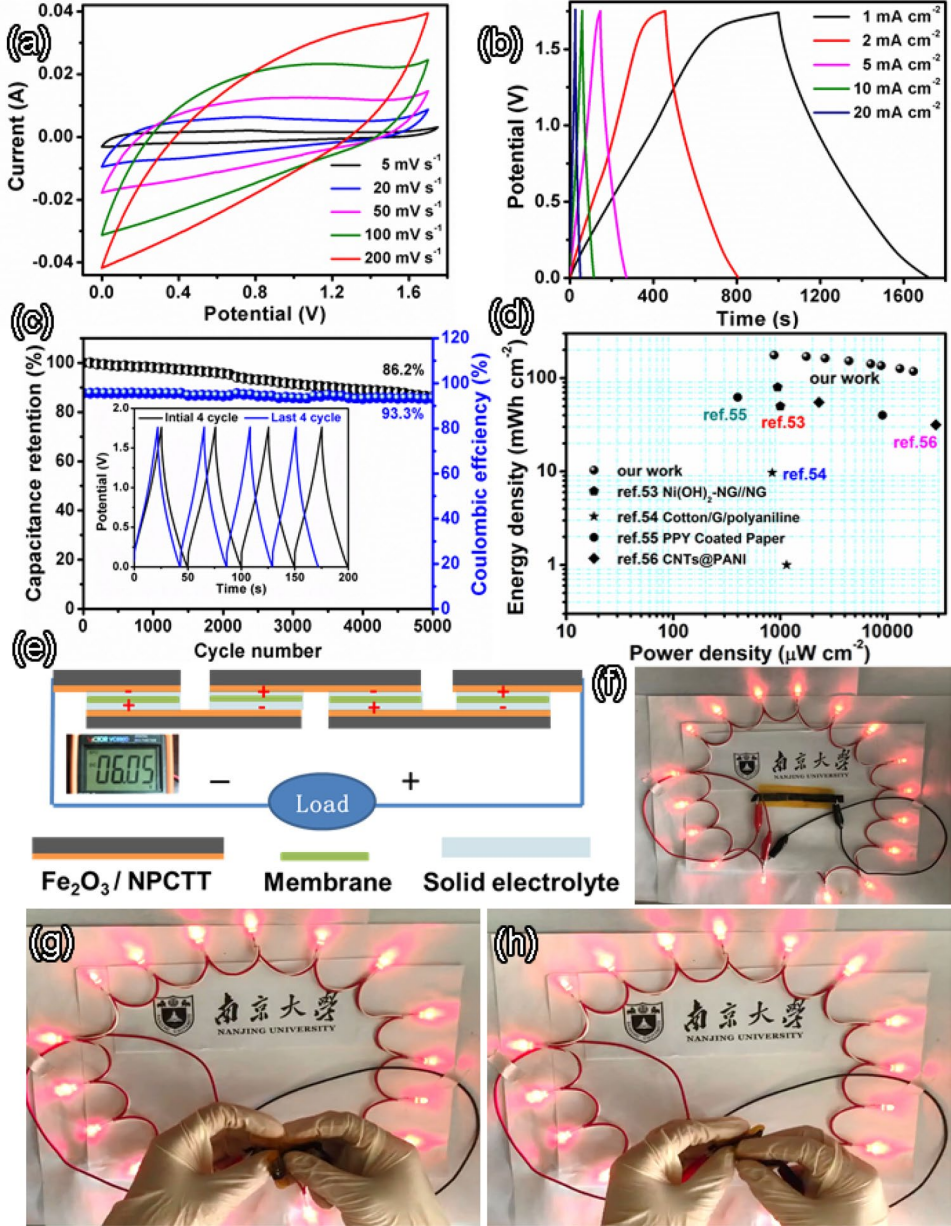
(**a**) CV curves at various scan rates, (**b**) GCD curves at different current densities of an solid-state symmetric supercapacitor with Fe_2_O_3_/NPCCT electrode, and (**c**) Cycling performance and Coulombic efficiency of the supercapacitor (measured at a high current density of 20 mA cm^−2^) with an inset showing the initial and last four cycles; (**d**) Ragone plots of our device with comparison to previous reports. (**e**) Schematic illustrations of assembled structure of a tandem by connecting four SCs in series; (**f**) Digital photos showing 18 LEDs powered by the tandem, and (**g**) the tandem being fully folded (bending angle 180°) and (**h**) being largely twisted.

A two-electrode symmetric solid-state supercapacitor using the Fe_2_O_3_/NPCCT was assembled. In comparison with the asymmetric SCs, symmetric devices have shorter charge–discharge time and more safer, and have no polarity due to same material as an anode and cathode which also prevent the catastrophic failure of device. Figure 7a shows CV curves at various scan rates of a symmetric supercapacitor. The CV curves have a wide operating voltage window 0–1.75 V, which is double of that for a single electrode, and wider than many reports for asymmetric SCs. Owing to the unique Fe_2_O_3_/NPCTT hybrid electrode and the use of PVA/KOH solid-state electrolyte, the symmetric device achieved a high voltage of 1.75 V. With a gradual increase of scan rate from 5 to 200 mV s^−1^, no obvious distortion of CV curves’ shape is observed. Figure 7b shows GCD curves of the symmetric device at various current densities, which reveals a reversible charge–discharge behavior. The potential range in the GCD curves is also 0–1.75 V, consistent with the CV curves. Although the symmetry of charging curve and discharging curve seem to be not good at a low current density of 1 mA cm^−2^ (the black curve), it is better at high current densities. Strikingly, even at a high current density of 20 mA cm^−2^, only 13.8% capacity decay is observed after 5,000 cycles, and the charge–discharge behavior becomes stable after the 4000th cycle (Fig. 7c), as demonstrated by comparison between the initial and the last four periods (see the inset). The Coulombic efficiency is 93.3% after 5,000 cycles, demonstrating remarkable reversibility and cycling stability of the device. The SC delivers a ultrahigh specific areal energy density of 176 μWh cm^−2^ at a power density of 875 μW cm^−2^. Even at a high power density of 17,500 μW cm^−2^, the device remains an energy density of 118 μWh cm^−2^, as shown in Ragone plot of Fig. 7d. To our knowledge, although asymmetric devices generally perform better than symmetric ones, the specific energy and power densities are larger than those of previously reported solid-state asymmetric devices such as Ni(OH)_2_@NG//NG (80 μWh cm^−2^ at 944 μW cm^−2^)^7^. Also, the energy density of 176 μWh cm^−2^ is much higher than most reported symmetric solid-state SCs based on composite electrode materials, such as cotton/graphene/polyaniline (cotton fibers with graphene sheets and polyaniline nanowire arrays, 9.74 μWh cm^−2^ at 840 μW cm^−2^)^53^, Polypyrrole coated air-laid paper (62.4 μWh cm^−2^ at 420 μW cm^−2^)^54^, and CNTs@PANI (50.98 μWh cm^−2^ at 2,294 μW cm^−2^)^55^, core-sheath graphene fibers (0.17 µWh cm^−2^)^56^, PPy/MO/Cotton fiber (7.5 µWh cm^−2^)^57^, CNT/ordered mesoporous carbon (1.77 µWh cm^−2^)^58^ and graphene/carbon nanotube core-sheath fibers (5.91 µWh cm^−2^)^59^, also see Table S2.

The applicability of solid-state supercapacitor device in practical conditions can be proved visibly by powering some small electronics. Figure 7e illustrates a scheme of assembled structure of a integrated tandem by connecting four symmetric SCs in series. The Fe_2_O_3_/NPCCT electrodes were placed interlaced together to form the solid KOH/PVA gel electrolyte films in the middle. The integrated tandem consisting of several connected SCs in series can obtain a desirable high-output-voltage, as confirmed by a tandem with a output voltage of 6.05 V (see the inset), which can power 18 LEDs (1.6 V, 5 mA) for a long time (Fig. 7f). Even the integrated tandem also has an outstanding flexibility and strong stability. The tandem has overlapped CV curves and negligible capacitance degradation at different twisting angles. In particular, in the case of being fully folded (face-to-face folding) with a bending angle of 180° and being large twisted, the tandem can keep the current output smoothly, and there are no structural failure and capacity loss during repeated large-angle twisting during discharging (Fig. 7g–h). This indicates that the device could be twisted arbitrarily almost without degrading its performance. The outstanding reliability promises many opportunities for a wide range of applications.

In summary, hierarchical and highly stable electrodes with Fe_2_O_3_ nanobelts arrays rooted in the surface layer of NPCTT were prepared by a proposed method of high-temperature carbonization of an alkali activated cotton cloth containing precursor. Modulation of the nanostructure leads to that the specific capacitance is maximized with a *C*_S_^2−^ = 20 mM. The unique composite structure of nanostructure arrays rooted in the surface layer of CT enables the Fe_2_O_3_/NPCCT electrodes with both good electrical conductivity and strong mechanical stability. Electrochemical performance of the resulting Fe_2_O_3_/NPCTT electrodes is optimized as a specific capacitance reaching 1846 mF cm^−2^ at a current density of 1 mA cm^−2^ and remarkable cycling stability. These are attributed to strong synergistic contribution of pseudo-capacitive Fe_2_O_3_ and EDLC NPCTT, highly porous but robust architecture of nanobelts arrays with carbon CTs as well as excellent electrical conductivity. Also, the Fe_2_O_3_/NPCCT electrodes have excellent flexibility and stretchability. A symmetric solid-state device with the Fe_2_O_3_/NPCCT delivers a ultrahigh specific areal energy density of 176 μWh cm^−2^ and shows outstanding reliability with no capacity degradation under repeated large-angle twisting. This work opens up a new route to high-performance flexible electrode for next-generation wearable electronics.

## Supplementary information

Supplementary file1 (PDF 1233 kb)
