# Supplementary material for: Iron oxides nanobelt arrays rooted in nanoporous surface of carbon tube textile as stretchable and robust electrodes for flexible supercapacitors with ultrahigh areal energy density and remarkable cycling-stability
Source: Sci Rep. 2020 Jul 3;10:11023. doi: 10.1038/s41598-020-68032-z (PMC7335107; doi:10.1038/s41598-020-68032-z)
Supplement: Supplementary file 1 — Supplementary file1 (PDF 1233 kb) [file 41598_2020_68032_MOESM1_ESM.pdf]

Supporting Information

Iron oxides nanobelt arrays rooted in nanoporous surface of carbon tube textile as stretchable and robust electrodes for flexible supercapacitors with ultrahigh areal energy density and remarkable cycling-stability

Yuying Ding, Shaochun Tang*, Rubing Han, Sheng Zhang, Guanjun Pan & Xiangkang Meng*

National Laboratory of Solid State Microstructures, Collaborative Innovation Center of Advanced Microstructures, Jiangsu Key Laboratory of Artificial Functional Materials, College of Engineering and Applied Sciences, Department of Materials Science & Engineering, Nanjing University, Jiangsu, 210093, P. R. China

* Correspondence and requests for materials should be addressed to S.C. Tang (tangsc@nju.edu.cn) or X.K. Meng (mengxk@nju.edu.cn).

Methods

Figure S1. TGA curve of recorded from the typical Fe_2O_3/NPCTT.

Figure S2. SEM images of a cotton textile before and after alkaline activation

Figure S3. SEM images showing the partly broken coating of grown nanostructures obtained from direct carbonization without alkali activation of cotton cloth.

Figure S4. SEM images of Fe_2O_3/NPCTT products obtained with different $C_{Fe}{}^{3+}$

Figure S5. High-resolution XPS spectrum of Mn 2p recorded from MnO/NPCTT

Figure S6. SEM images and SEM-mappings of typical Co_3O_4/NPCTT product

Figure S7. GCD curves of the electrodes obtained with different $C_{Fe}{}^{3+}$

Figure S8. SEM images showing the Fe_2O_3/NPCCT after cycling measurements

Table S1. Electrochemical performance comparison with reported values

Table S2. The energy density comparison of our SCs with reported values

Methods

Synthesis of Fe_2O_3-nanobelt arrays/NPCTT

All chemical reagents were analytical grade, and used without further purification. A piece of cotton textile with a size of 3 cm length × 1 cm width was cut from a commercially available white cotton T-shirt containing 100% cotton. To start with, the cotton textile was pre-treated in 100 ml NaOH solution with a concentration of 1 M under a continuous ultrasound irradiation at a power of 100 W for 1 h. Then, it was vacuum dried at 80 ℃ and a dried alkali activated cotton textile was obtained.

As for the synthesis of Fe_2O_3-nanobelt arrays/NPCTT, typically, 808 mg $Fe(NO_3)_2 \cdot 9H_2O$ were dissolved in 100 ml of deionized water, leading to an aqueous solution with Fe^{3+} concentration of 20 mM. After that, the dried alkali activated cotton textile was immersed in the Fe^{3+} solution. After continuous ultrasound irradiation at 100 W for 10 min, the textile was taken out and vacuum dried again with the same drying conditions. The treated cotton textile was put at the center position of a tube furnace, and heated for 1 h at a constant temperature of 800 ℃ with continuous flow of Ar gas (high-temperature carbonization). Fe_2O_3 nanobelt arrays rooted in surface layer of NPCTT were obtained during the high-temperature carbonization process. To investigate the influence of Fe^{3+} concentration on the resulting products, we also performed control experiments with varying Fe^{3+} concentrations such as 5 mM, 10 mM, 30 mM and 50 mM.

Synthesis of MnO nanosheet arrays/NPCTT composite was performed by the same procedures as mentioned above, similar to that of Fe_2O_3-nanobelt arrays/NPCTT. Typically, an aqueous $MnSO_4$ solution with a concentration of 10 mM was used instead of 20 mM $Fe(NO_3)_2$, while other conditions were remained the same. Co_3O_4

nanowire arrays/NPCTT. Similarly, the synthesis of Co_3O_4 nanowire arrays/NPCTT was carried out by using 50 mM of an aqueous $CoCl_2$ solution instead.

Materials characterizations

The products were characterized by X-ray diffraction (XRD) with a D/Max-RA X-ray diffractometer (Cu Kα =1.5418 Å radiation) at 5° min^{-1}. Morphology and size were analyzed by field-emission scanning electron microscopy (FE-SEM) on S-4800 (Hitachi) at 10 kV. Transmission electron microscopy (TEM) and high-resolution (HRTEM) images were recorded on a JEM-2100 TEM (JEOL, 200 kV). Since the wall thickness of a single micron carbon tube (core) is about 1 μm, and the grown nanobelts have lengths larger than 5 μm, it is very difficult to perform TEM characterization because electron beam cannot transmit such thick object. In order to perform TEM, we scraped the Fe_2O_3 off the surface of CTs, followed by ultrasonic dispersion of them in alcohol. X-ray photoelectron spectroscopy (XPS) analyzed the valence states of elements in a Thermo VG Scientific MultiLab ESCA2000 system with a CLAM4 hemispherical analyzer at a base pressure below 3×10^{-10} mbar. The energy-dispersed X-ray spectroscopy (EDS), SEM-EDS, SEM-mapping analysis were performed on the same FE-SEM microscope. X-ray photoelectron spectroscopy (XPS) was analyzed in a Thermo VG Scientific MultiLab ESCA2000 system with a CLAM4 hemispherical analyzer at a base pressure below 3×10^{-10} mbar. Thermogravimetric analysis (TGA) tests were performed by using a TGA/SDTA thermogravimetric analyzer from room temperature to 800℃ in air at a heating rate of 10 ^oC min^{-1}.

Electrochemical measurements

All the electrochemical experiments were carried out on a Metrohm Autolab 302N electrochemical workstation. In a three-electrode measurement system, the

prepared Fe_2O_3/NPCTT was used as a working electrode (1 cm^2 immersed area), a platinum plate served as the counter electrode, and an Ag/AgCl electrode as the reference, all in 3 M KOH aqueous electrolyte. Cyclic voltammetry (CV) was recorded from 0 to 0.8 V (vs. Ag/AgCl) at various scan rates. Galvanostatic charge-discharge (GCD) tests were carried out at different current densities. Specific areal capacitances (F cm^{-2}) were calculated from the discharge curves via $C_a = (I\ \Delta t) / (S\ \Delta V)$, where I is the discharge current, S is an effective area, Δt is discharge time, and ΔV is the voltage change due to discharging. Cycling stability tests were carried out at a constant current density for 10 thousand cycles on a Land Battery Test System (Wuhan Kingnuo Electronic Company, China). The capacitance retention of any cycle equals its discharge time divided by the discharge time of the first cycle. Coulombic efficiency is calculated from an equation $\eta = t_d / t_c \times 100\%$, where t_c and t_d are the charge and discharge times. In the electrochemical impedance spectroscopy (EIS) measurements, AC amplitude was 5 mV and frequency ranges from 10^5 to 0.1 Hz.

In order to assemble two-electrode solid-state SCs, a filter paper based solid electrolyte film was prepared at first. Typically, 1 g of KOH and 1.5 g of PVA powder were added into 18 ml of deionized. The mixture was then heated to 90 ^oC under violent stir. The solution was mechanically stirred slowly for another 2 h at r.t. in order to eliminate bubbles. Finally, the solution was dripped onto two sides of a filter paper and dried in air to form KOH/PVA gel membranes. The solid electrolyte film was sandwiched in between two electrodes and thus works as a solid electrolyte and separator. CV and GCD measurements of solid-state **SCs** were performed on the same electrochemical workstation. Long-term cycling stability was also tested on a the Land Battery Test System. Specific energy density E and power density P are defined as $E = (1/2)\ C\ (\Delta V)^2$ and $P = E / \Delta t$.

Fig. S1

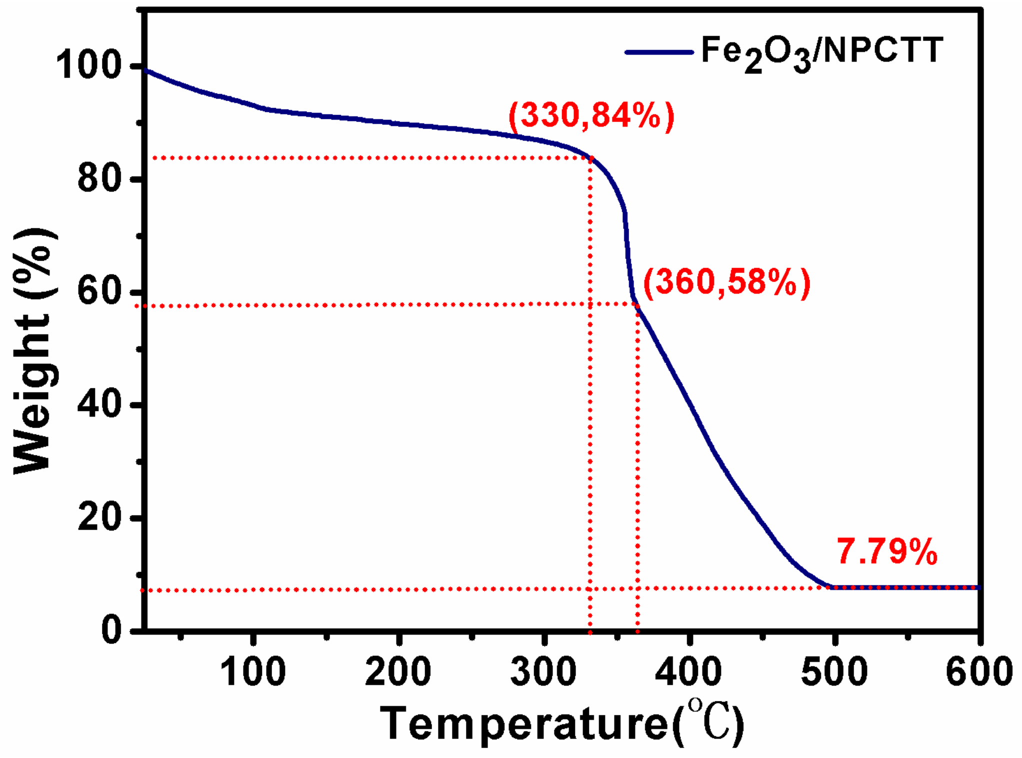

Figure S1. TGA curve of recorded from the typical Fe_2O_3/NPCTT.

Fig. S2

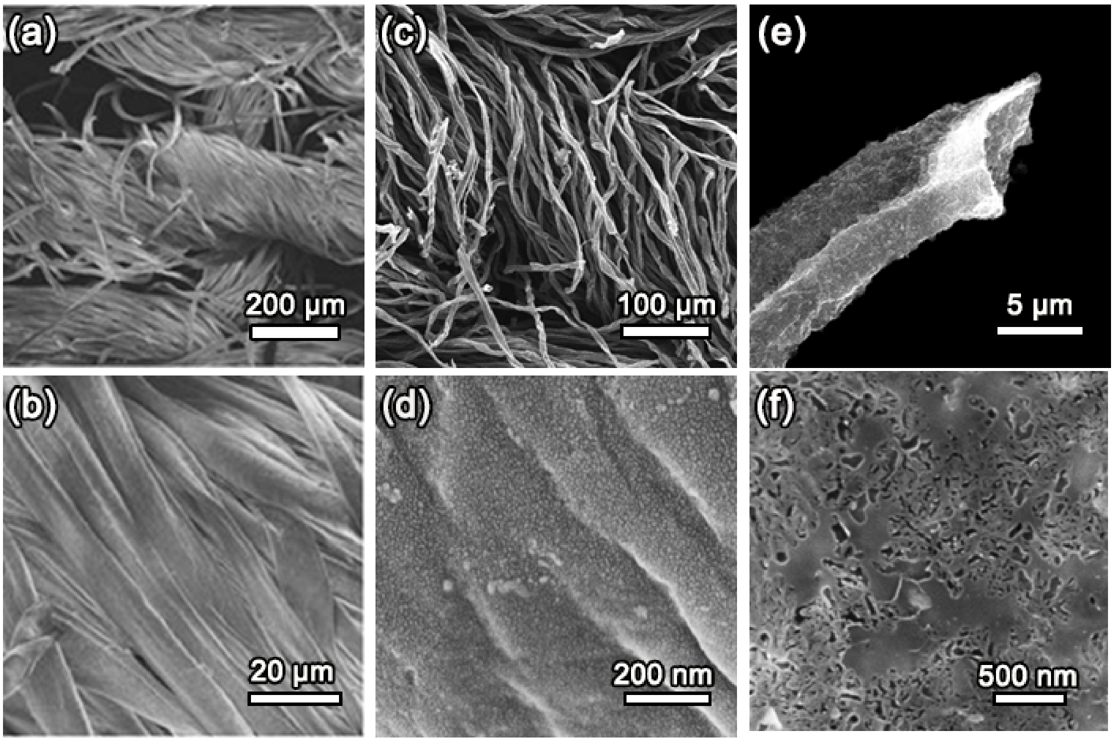

Figure S2. (a) low- and (b) high-magnification SEM images of a pristine cotton textile before and (c, d) after alkaline activation; (e) low- and (f) magnified SEM images of the resulting a single alkali activated carbon tube in a NPCTT.

Fig. S3

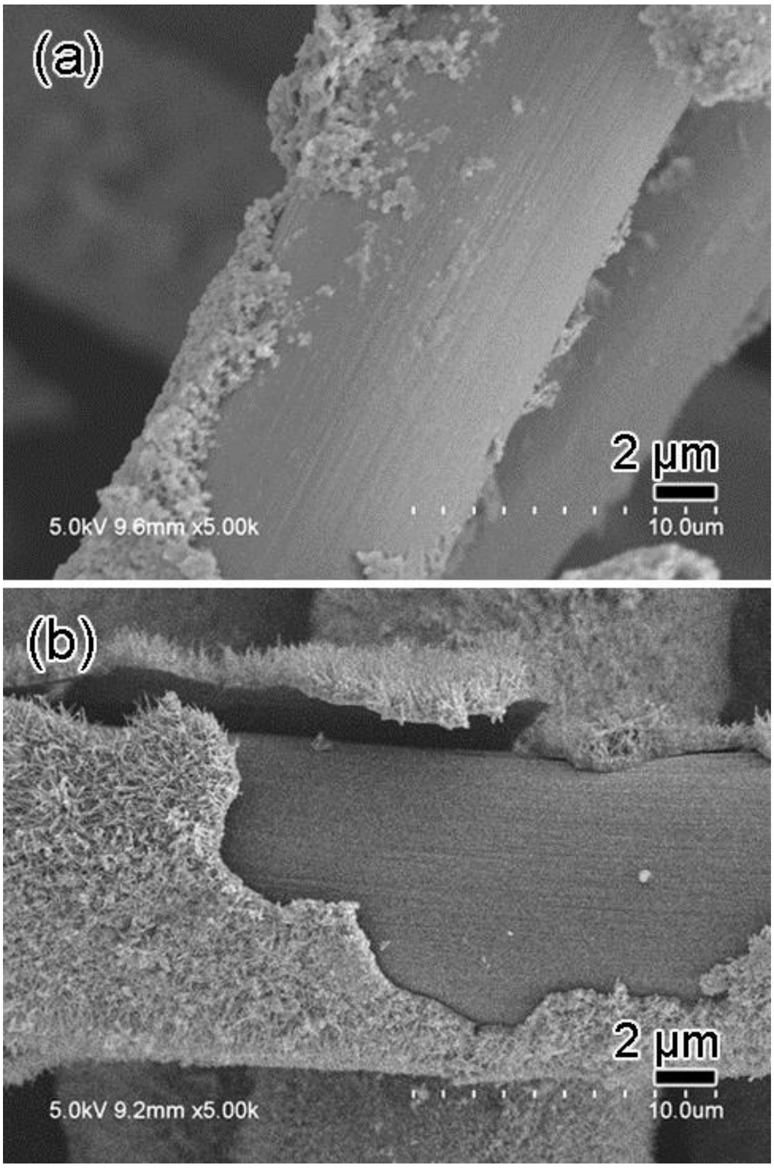

Figure S3. SEM images showing the partly broken coating of grown nanostructures on a single carbon tube in a carbon cloth obtained from direct high-temperature carbonization without alkali activation of cotton cloth. (a) nanoparticle coating. (b) a uniform coating of nanowire arrays after ultrasound irradiation for only 10 min at a power of 100 W.

Fig. S4

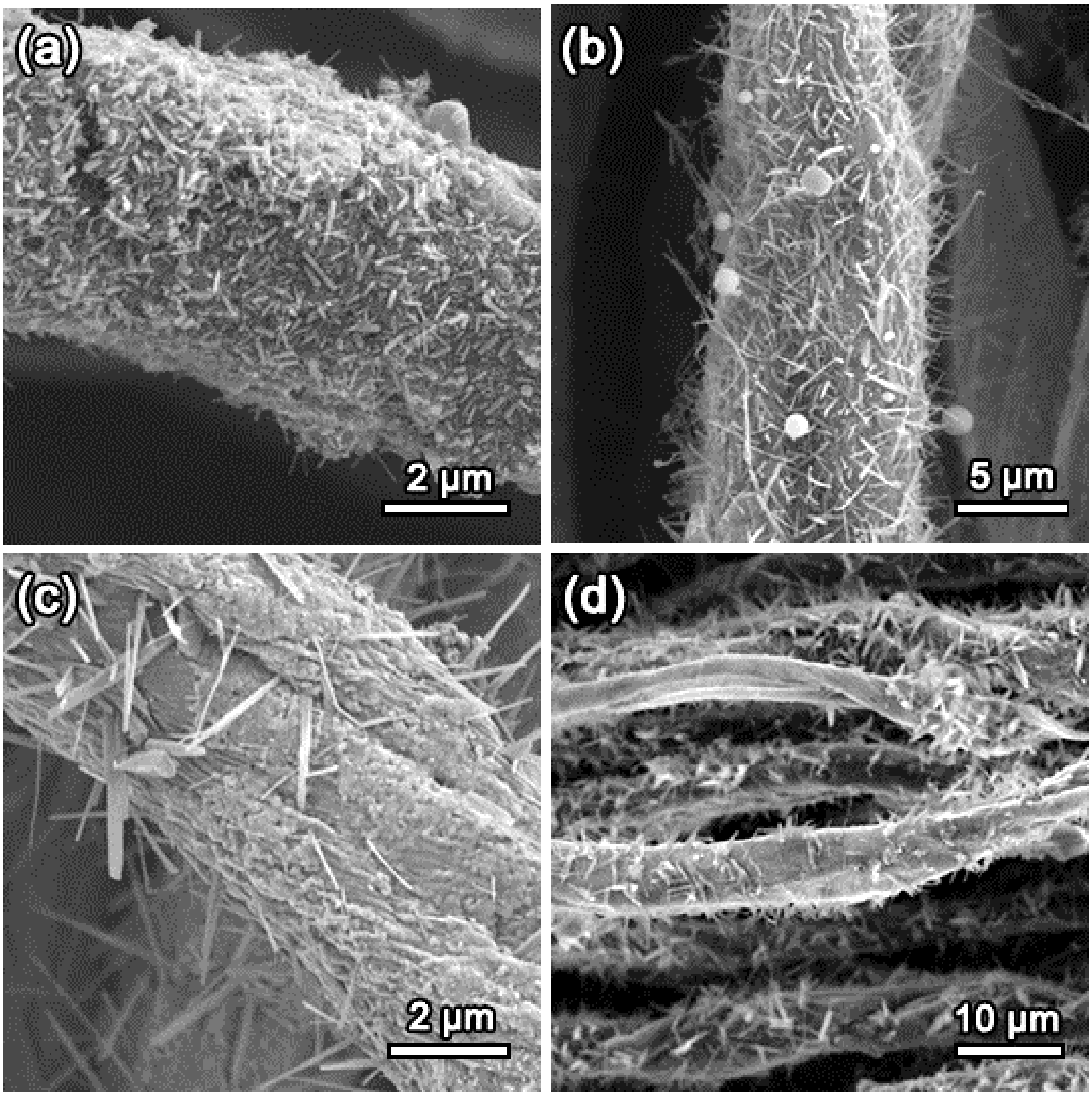

Figure S4. SEM images of Fe_2O_3/NPCTT products obtained with different $C_{Fe}{}^{3+}$ of (a) 5 mM, (b) 10 mM, (c) 30 mM and (d) 50 mM.

Fig. S5

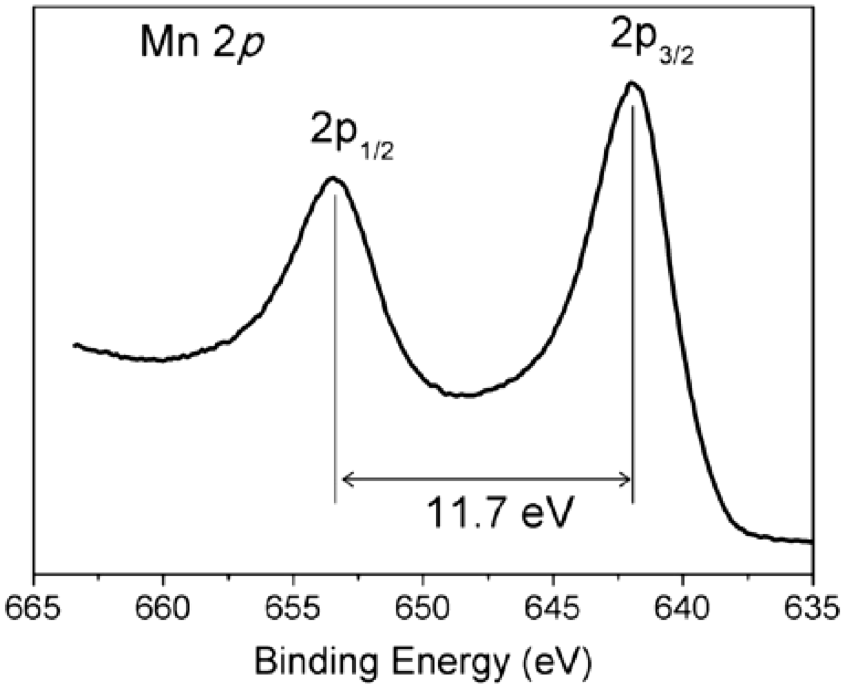

Figure S5. High-resolution XPS spectrum of Mn 2*p* recorded from MnO/NPCTT.

Fig. S6

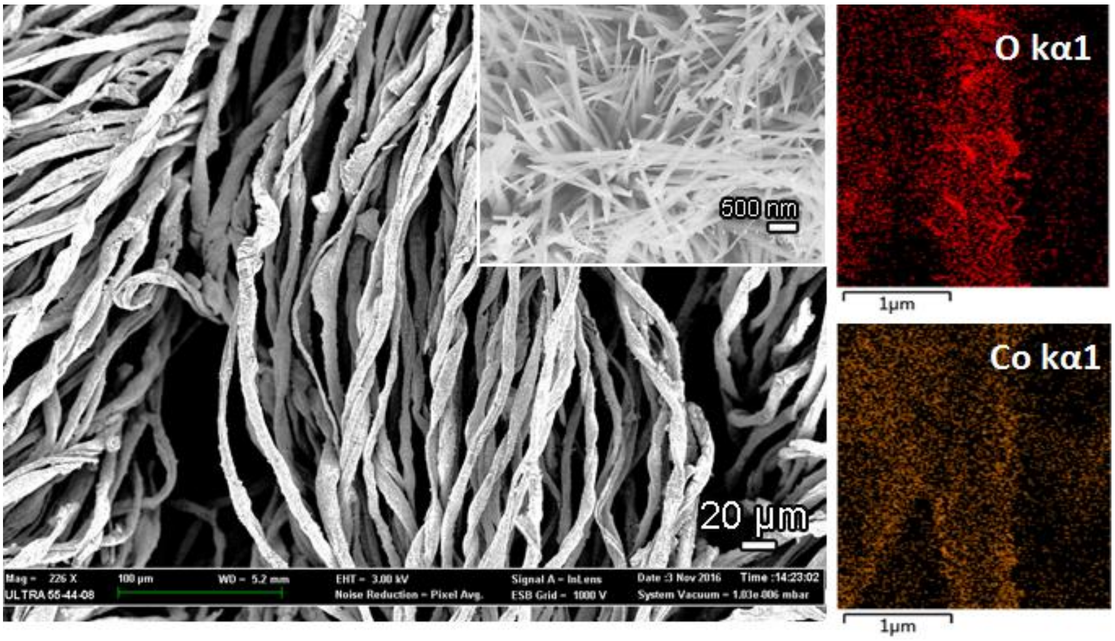

Figure S6. (left) A SEM image with an inset showing typical Co3O4/NPCTT product. (right) SEM-mapping of elemental O and Co distribution along a single coated CT.

Fig. S7

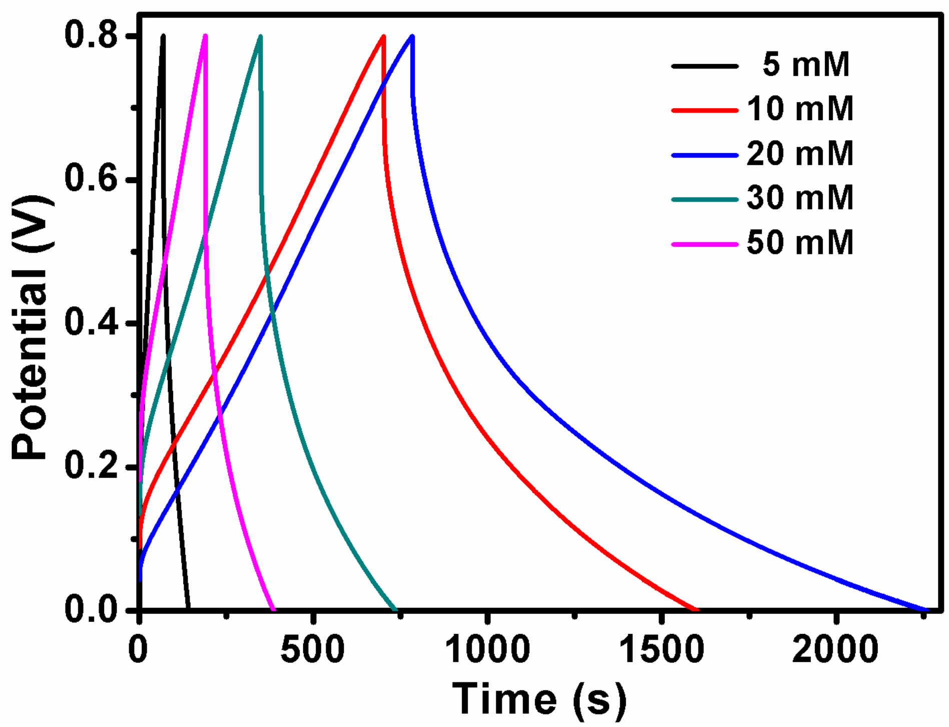

Figure S7. The corresponding GCD curves (all at 1 mA cm^{-2}) of the electrodes obtained with different $C_{Fe^{3+}}$.

Fig. S8

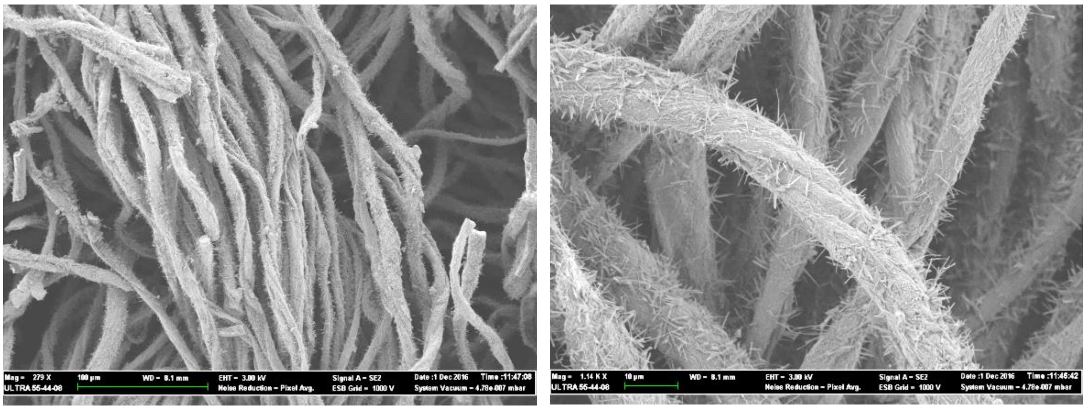

Figure S8. SEM images showing the Fe_2O_3/NPCCT electrode after the cycling measurements, the Fe_2O_3 nanobelt arrays were still well attached on the carbon tubes.

Table S1. **Electrochemical performance comparison of our Fe_2O_3 nanobelt arrays/NPCTT electrode with reported values in the literature.**

Electrode materials	Specific capacitances (Current density)	Capacitance retention (Cycle number)	References
Fe_2O_3 nanobelt arrays/NPCTT	**1846** mF cm^{-2} (1 mA cm^{-2})	**95.2% (10,000)**	**Our work**
Commercially available carbon fiber cloth	**13** mF cm^{-2} (1 mA cm^{-2})	/	**44**
Molten-$NaNH_2$ activated carbon cloth	**744.5** mF cm^{-2} (1 mA cm^{-2})	**96.4% (10,000)**	**45**
N-doped carbon nanotubes	**180** mF cm^{-2} (0.5 mA cm^{-2})	**95% (5,000)**	**46**
N-doped carbon cloth	**136** mF cm^{-2} (0.5 mA cm^{-2})	/	**47**
Graphene-wrapped Fe_2O_3 nanowire networks	**3.3** mF cm^{-2} (10 mA cm^{-2})	**78.2% (5,000)**	**48**
Fe_2O_3 nanorods@Graphene foam on Ni foam	**572** mF cm^{-2} (1 mA cm^{-2})	/	**49**
Fe_2O_3 nanorods@NiO nanosheets on carbon cloth	**557** mF cm^{-2} (1 mA cm^{-2})	**96.2% (3,000)**	**15**
Graphene foam-CNT@ Fe_2O_3 nanoparticles	**470.5** mF cm^{-2} (1 mA cm^{-2})	**93.5% (50000)**	**50**

Table S2 **The energy density comparison of our SCs with reported values.**

All-solid-state SCs	Power density (μW cm^{-2})	Energy density (μWh cm^{-2})	References
**Fe_2O_3/NPCTT // Fe_2O_3/NPCTT**	875	176	**Our work**
**$Ni(OH)_2$-NG // NG**	944	80	**53**
**CT/G/PANI // CT/G/PANI**	840.9	9.7	**54**
**PPY air-laid paper // PPY air-laid paper**	400	62.4	**55**
**CNT@PANI // CNT@PANI**	2294	50.94	**56**
**Core-sheath graphene fibers**	100	0.17	**57**
**PPy nanotubes coated cotton**	/	7.5	**58**
**CNT/ordered mesoporous carbon composite fibers**	/	1.77	**59**
**G/CNT core-sheath fibers**	20	3.84	**60**

NG: N-doped graphene
CT/G/PANI: cotton/graphene/polyaniline
PPY: Polypyrrole
CNT: carbon nanotube
